# Feminizing *Wolbachia* endosymbiont disrupts maternal sex chromosome inheritance in a butterfly species

**DOI:** 10.1002/evl3.28

**Published:** 2017-10-31

**Authors:** Daisuke Kageyama, Mizuki Ohno, Tatsushi Sasaki, Atsuo Yoshido, Tatsuro Konagaya, Akiya Jouraku, Seigo Kuwazaki, Hiroyuki Kanamori, Yuichi Katayose, Satoko Narita, Mai Miyata, Markus Riegler, Ken Sahara

**Affiliations:** ^1^ Institute of Agrobiological Sciences National Agriculture and Food Research Organization Tsukuba Ibaraki 305–0854 Japan; ^2^ Laboratory of Applied Entomology, Faculty of Agriculture Iwate University Morioka 020–8550 Japan; ^3^ Graduate School of Science Kyoto University Kyoto 606–8502 Japan; ^4^ Institute of Crop Science National Agriculture and Food Research Organization Tsukuba Ibaraki 305–0854 Japan; ^5^ Tsukuba Primate Research Center National Institute of Biomedical Innovation Hachimandai Tsukuba Ibaraki 305–0843 Japan; ^6^ Graduate School of Horticulture Chiba University Matsudo Chiba 271–8510 Japan; ^7^ Hawkesbury Institute for the Environment Western Sydney University Penrith New South Wales 2751 Australia

**Keywords:** Butterfly, chromosome inheritance, sex determination, *Wolbachia*

## Abstract

*Wolbachia* is a maternally inherited ubiquitous endosymbiotic bacterium of arthropods that displays a diverse repertoire of host reproductive manipulations. For the first time, we demonstrate that *Wolbachia* manipulates sex chromosome inheritance in a sexually reproducing insect. *Eurema mandarina* butterfly females on Tanegashima Island, Japan, are infected with the *w*Fem *Wolbachia* strain and produce all‐female offspring, while antibiotic treatment results in male offspring. Fluorescence in situ hybridization (FISH) revealed that *w*Fem‐positive and *w*Fem‐negative females have Z0 and WZ sex chromosome sets, respectively, demonstrating the predicted absence of the W chromosome in *w*Fem‐infected lineages. Genomic quantitative polymerase chain reaction (qPCR) analysis showed that *w*Fem‐positive females lay only Z0 eggs that carry a paternal Z, whereas females from lineages that are naturally *w*Fem‐negative lay both WZ and ZZ eggs. In contrast, antibiotic treatment of adult *w*Fem females resulted in the production of Z0 and ZZ eggs, suggesting that this *Wolbachia* strain can disrupt the maternal inheritance of Z chromosomes. Moreover, most male offspring produced by antibiotic‐treated *w*Fem females had a ZZ karyotype, implying reduced survival of Z0 individuals in the absence of feminizing effects of *Wolbachia*. Antibiotic treatment of *w*Fem‐infected larvae induced male‐specific splicing of the *doublesex* (*dsx*) gene transcript, causing an intersex phenotype. Thus, the absence of the female‐determining W chromosome in Z0 individuals is functionally compensated by *Wolbachia*‐mediated conversion of sex determination. We discuss how *Wolbachia* may manipulate the host chromosome inheritance and that *Wolbachia* may have acquired this coordinated dual mode of reproductive manipulation first by the evolution of female‐determining function and then cytoplasmically induced disruption of sex chromosome inheritance.

**Impact Summary**

Genomes are vulnerable to selfish genetic elements that enhance their own transmission often at the expense of host fitness. Examples are cytoplasmic elements such as maternally inherited bacteria that cause feminization, male‐killing, parthenogenesis, and cytoplasmic incompatibility. We demonstrate, for the first time, that the inheritance of a chromosome can be hampered by the ubiquitous endosymbiotic bacterium *Wolbachia*. For *Eurema mandarina* butterfly lineages with a Z0 sex chromosome constitution, we provide direct and conclusive evidence that *Wolbachia* induces production of all‐female progeny by a dual role: the compensation for the female‐determining function that is absent in Z0 lineages and the prevention of maternal sex chromosome inheritance to offspring. Therefore, our findings highlight that both sex determination and chromosome inheritance—crucially important developmental processes of higher eukaryotes—can be manipulated by cytoplasmic parasites.

Genomes of sexually reproducing organisms are exposed to genetic conflicts. For example, some genes bias reproduction toward male offspring while other genes within the same genome may favor reproduction of more daughters. Selfish genetic elements (SGEs), such as meiotic drivers, cytoplasmic sex ratio distorters and transposons, are extreme examples, which enhance their own transmission often at the expense of their hosts’ fitness (Burt and Trivers 2006; Werren 2011). There is growing evidence that SGEs, and their genetic conflict with host genomes, trigger important evolutionary change and innovation in eukaryotes (Werren 2011).

Meiotic drive, also referred to as segregation distortion (SD), is a violation of Mendelian law as it leads to the more frequent inheritance of one copy of a gene than the expected 50% (Jaenike 2001; Lindholm et al. 2016). A segregation distorter that sits on a sex chromosome biases the sex ratio. For example, X‐linked segregation distorter (X drive) and Y‐linked segregation distorter (Y drive) in flies (Diptera), result in female‐biased and male‐biased sex ratios, respectively (Lindholm et al. 2016). In male‐heterogametic species, X and Y drivers are expected to be encoded in the nuclear genome. In female‐heterogametic species, however, W chromosome and cytoplasm behave as a single linkage group and thus distortion of sex chromosome inheritance in female‐heterogametic species can theoretically also be caused by cytoplasmic elements. Although this possibility has previously been proposed (Hurst 1993; Beukeboom and Perrin 2014), lack of empirical evidence questions whether it is mechanistically possible for cytoplasmic elements to cause meiotic drive.

*Wolbachia pipientis* (Alphaproteobacteria), simply referred to as *Wolbachia*, attracts significant interest in evolutionary and developmental biology but also in applied fields such as pest management because it can manipulate reproduction of arthropods in various ways such as cytoplasmic incompatibility, parthenogenesis induction, feminization, and male‐killing (Werren et al. 2008). Here we demonstrate for the first time that *Wolbachia* is responsible for the disruption of sex chromosome inheritance. We do this by providing multifaceted and conclusive evidence that in the butterfly *Eurema mandarina Wolbachia*‐induced disruption of chromosome inheritance, which may be the result of SD, constitutes the underlying mechanism for the production of all‐female progeny. In most populations, *E. mandarina* is infected with the cytoplasmic‐incompatibility (CI)‐inducing *Wolbachia* strain *w*CI at a high prevalence of close to 100% (Hiroki et al. 2005; Narita et al. 2006). Hiroki et al. (2002 and 2004) first reported all‐female offspring production in *E. mandarina* (then known as *Eurema hecabe* yellow type), which was considered to be due to the feminization of genetic males (ZZ) by coinfections with the *Wolbachia* strain *w*Fem (hereafter referred to as double infection CF while single infection with *w*CI is referred to as C). Three observations about CF lineages supported this view, that is (a) antibiotic treatment of adult females led to the production of all‐male offspring (Hiroki et al. 2002), (b) antibiotic treatment of larvae resulted in intersex adults (Narita et al. 2007a) and (c) females did not have the W chromatin body (Hiroki et al. 2002; Narita et al. 2007a). This has recently been challenged, because it was demonstrated that CF females have only one Z chromosome and that this Z chromosome always derived from their fathers implying that a disruption of chromosome inheritance may be in place although it was not clear whether *Wolbachia* induced the disruption (Kern et al. 2015). As a consequence, two novel (yet untested) hypotheses were formed, namely, that CF females have either a Z0 or a W'Z sex chromosome set (whereby W’ cannot be visualized in W chromatin assays and does not have a female‐determining function), and that the disruption of Z chromosome inheritance occurs in CF lineages due to *Wolbachia* or another factor, such as those encoded by the host nucleus. Moreover, the intensity of chromosome disruption was not known, and therefore, killing of ZZ males may complement the incomplete chromosome disruption to achieve all‐female production.

In a multifaceted approach, by combining fluorescence in situ hybridization (FISH), genome sequencing, quantitative PCR, reverse transcription PCR and antibiotic treatment, we have tested these hypotheses and revealed that CF females have Z0, and that *Wolbachia* is the cause for both the 100% disruption of Z chromosome inheritance and the female sex determination of Z0 individuals. Our results demonstrate, for the first time, *Wolbachia* as the agent that is responsible for distorted sex chromosome inheritance, and thereby highlight that cytoplasmic elements can have profound effects on oogenesis, sex chromosome inheritance, and sex determination–fundamental biological processes of eukaryotes.

## Methods

### COLLECTION AND REARING OF *E. MANDARINA*

Female adults of *E. mandarina* (Lepidoptera: Pieridae) were collected on Tanegashima Island, Kagoshima, Japan (Fig. S1). In the laboratory, each female was allowed to lay embryos on fresh leaves of *Lespedeza cuneata* (Fabales: Fabaceae) in a plastic cup with absorbent cotton immersed with 5% honey solution. The artificial diet for larvae was prepared by mixing leaf powder of *Albizia julibrissin* (Fabales: Fabaceae) in the custom‐made Silkmate devoid of mulberry leaves (Nihon‐Nosan, Yokohama, Japan). Insects were reared under the 16 h/8 h light/dark photoperiod at 25°C.

### ANTIBIOTIC TREATMENT

We performed antibiotic treatment of two different stages (larval stage and adult stage) of *E. mandarina*. For larval antibiotic treatment, larvae were fed with the artificial diet (shown above) containing 0.05% tetracycline hydrochloride (tet). For adult antibiotic treatment, female adults were fed with 5% honey solution containing 0.1% tet. Specifically, CF females were mated to antibiotic‐treated male offspring of C females. Antibiotic treatment of these males was to avoid a possible occurrence of CI. After mating, each CF female was allowed to lay eggs on fresh leaves of *L. cuneata* in a plastic cup with absorbent cotton immersed with 5% honey solution containing 0.1% tet. Fresh leaves of *L. cuneata* and cotton with tet‐containing honey solution were exchanged daily.

### DIAGNOSIS OF *WOLBACHIA* STRAINS

To diagnose *Wolbachia* strains in *E. mandarina*, several legs of each adult were homogenized in STE buffer (10 mM Tris‐HCl (pH 8.0), 1 mM EDTA (pH 8.0), 150 mM NaCl) and incubated at 56°C for 30 min followed by 92°C for 5 min. After centrifugation at 15,000 rpm for 2 min, the supernatant was used for polymerase chain reaction (PCR) using different primer pairs. The primer pair wsp81F (5′–TGGTCCAATAAGTGATGAAGAAAC–3′) and wsp691R (5′–AAAAATTAAACGCTACTCCA–3′) amplifies a ca. 610‐bp fragment of the *Wolbachia wsp* gene (Braig et al. 1998). The primer pair wsp81F and HecCIR (5′–ACTAACGTCGTTTTTGTTTAG–3′) amplifies a 232‐bp fragment of the *wsp* gene of *w*CI, while the primer pair HecFemF (5′–TTACTCACAATTGGCTAAAGAT–3′) and the wsp691R amplifies a 398‐bp fragment of *wsp* gene of *w*Fem (Hiroki et al. 2004; Narita et al. 2007b).

### WHOLE GENOME SEQUENCING AND *DE NOVO* ASSEMBLY

We performed whole genome sequencing for three types of *E. mandarina* individuals (CF females, C females, and C males) that were collected on Tanegashima Island, Japan (Fig. S1). Six genomic DNA libraries (two libraries for each sample type derived from two individuals) were constructed following manufacturer's instructions (http://www.illumina.com). The average insert size of the libraries was approximately 350 bp and each library was multiplexed using a single indexing protocol. The genomic DNA libraries were sequenced by Illumina MiSeq using MiSeq Reagent Kit v3 (600‐cycle) (Illumina, San Diego, CA). Generated raw reads (8.31 Gb, 5.34 Gb, and 6.94 Gb for CF females, C females and C males, respectively) were filtered by Trimmomatic (Bolger et al. 2014) and then mapped to the complete genome of *Wolbachia* strain *w*Pip (GenBank: NC_010981.1) by Bowtie2 (Langmead and Salzberg 2012). Mapped reads were discarded (to eliminate *Wolbachia* sequences) and then remaining reads of the three samples were merged and *de novo* assembled by SGA assembler (Simpson and Durbin 2012). Generated genome contig sequences were used for further analysis.

### ANALYSIS OF MAPPED READ COUNTS ON CHROMOSOMES

To verify that CF and C females have one Z chromosome, we compared normalized mapped read counts of the three samples on Z chromosomes and remaining chromosomes. The filtered reads of each sample were mapped to the genome contigs by Bowtie2 (only concordantly and uniquely mapped reads were counted) and then normalized mapped read count of each sample on each contig was calculated based on the ratio of the number of total mapped reads between the three samples. Nucleotide sequences of relatively long genome contigs (length is 2 kb or more) with enough coverage (20 or more mapped reads) were extracted and compared with the gene set A of *B. mori* (Suetsugu et al. 2013) by blastx search (cutoff e‐value is 1e‐50). Genome contigs with blastx hits were extracted and classified into 28 chromosomes based on the location of the homologous *B. mori* genes. For each chromosome, the average number of relative normalized mapped read counts was calculated for each sample (the number of C males was normalized to 1) using the normalized mapped read counts in the classified genome contigs, respectively.

### SANGER SEQUENCING

To genotype Z chromosomes, a highly variable intron of Z‐linked triosephosphate isomerase (*Tpi*) gene was PCR amplified using the primers, 5′–GGTCACTCTGAAAGGAGAACCACTTT–3′ and 5′–CACAACATTTGCCCAGTTGTTGCAA–3′, located in coding regions (Jiggins et al. 2001). The PCR products were treated with ExoSAP‐IT^®^ (Affymetrix Inc., Santa Clara, CA) and subjected to direct sequencing at Eurofins Genomics K.K. (Tokyo, Japan). No indels or SNPs were observed in sequence chromatograms of females; some males were heterozygous due to detected double peaks and shifts of sequence reads. By sequencing from both sides, it was possible to obtain the genotypes of males and females (Fig. S4).

### FISH ANALYSIS

In most lepidopteran species, a conspicuous heterochromatic body is exclusively found in female polyploid nuclei. Since W derived‐BAC as well as genomic probes have highlighted the W chromosomes and heterochromatin bodies in *B. mori* (Sahara et al. 2003a,b), there is no doubt that the bodies consist of the W chromosomes. The diagnosis however remains unreliable if a species of interest carries a W–autosomal translocation and/or partial deletion of the W (Traut and Marec 1996; Abe et al. 2008). Hiroki et al. (2002) as well as Narita et al. (2007a) relied on the W‐body diagnosis for C and CF females and concluded that they have WZ and ZZ sex chromosome constitutions, respectively. However, Kern et al. (2015) has recently found that, on the basis of genomic qPCR designed to amplify Z‐linked gene sequences (*Tpi* and *Ket*) relative to an autosomal gene (*EF‐1α*), both CF and C females have only one Z chromosome while males have two Z chromosomes. This finding rejected the previous conclusion that the sex chromosome constitution of CF females is ZZ (Hiroki et al. 2002; Narita et al. 2007a) but was inconclusive about whether CF females have a Z0 or W'Z system (with W’ as a modified W that has lost the female‐determining function and cannot be detected by the W‐body assay). Hence, we carried out more extensive chromosome analysis (other than just the W‐body) to directly prove whether CF females carry the W or not.

In Lepidoptera, the W chromosome can be highlighted by FISH using probes prepared from whole genomic DNA of males or females. The capability of FISH probes in detecting the W chromosome is due to the numerous repetitive short sequences occupying the W chromosome, which is then prone to be hybridized by random sequences. Genomic probes also paint repetitive regions scattered across other chromosomes, albeit at a lower density (autosomes and Z chromosome). Here we made mitotic and pachytene chromosome preparations from wing discs and gonads, respectively, in the last instar larvae of C and CF individuals of *E. mandarina* (see Yoshido et al. (2014) for details). Genomic DNA was extracted from tet‐treated C female larvae. Insect telomeric repeats were amplified by nontemplate PCR (Sahara et al. 1999). *Kettin* (*Ket*) gene fragments were amplified from adult cDNA synthesized by PrimeScript™ RT reagent Kit (TaKaRa, Otsu, Japan) and cloned by TOPO^®^ TA Cloning^®^ Kit (Thermo Fisher Scientific, Waltham, MA). We used four pairs of primers, Em_kettin_F1: 5′–AGGTAATCCAACGCCAGTCG–3′ and Em_kettin_R1: 5′–TGCTTGCCCTAAGGCATTGT–3′, Em_kettin_F2: 5′–ACAATGCCTTAGGGCAAGCA–3′ and Em_kettin_R2: 5′–TGGGCAAAGCCTCTTCATGT–3′, Em_kettin_F3: 5′–AGATTCCGCACTACGCATGA–3′ and Em_kettin_R3: 5′–TAAATTGTGGTGGGACGGCA–3′, Em_kettin_F5: 5′–ACATGAAGAGGCTTTGCCCA–3′ and Em_kettin_R5: 5′–TCATGCGTAGTGCGGAATCT–3′, for PCR amplification with 94°C for 5 min followed by 35 cycles of 94°C for 30 s, 60°C for 30 s and 72°C for 3 min finalized by 72°C for 10 min. Probe labeling was done by using the Nick Translation Kit (Abbott Molecular, Des Plaines, IL). We selected Green‐dUTP, Orange‐dUTP (Abbott Molecular Inc.) and Cy5‐dUTP (GE Healthcare Japan, Tokyo) fluorochromes for genomic DNA, *Ket* and insect telomeric repeat (TTAGG)*n* probes respectively. Hybridizations were carried out according to protocols described elsewhere (Yoshido et al. 2014). Signal and chromosome images were captured with a DFC350FX CCD camera mounted on a DM 6000B microscope (Leica Microsystems Japan, Tokyo) and processed with Adobe Photoshop CS2. We applied green, red and yellow pseudocolors to signals from Green, Orange, and Cy5, respectively.

### QUANTITATIVE POLYMERASE CHAIN REACTION (QPCR)

Embryos of mated females were sampled 48 h after the oviposition and stored at –80°C until DNA extraction. Embryos were individually subjected to DNA extraction using DNeasy^®^ Blood & Tissue Kit (Qiagen, Tokyo, Japan). Real‐time fluorescence detection quantitative PCR (qPCR) was performed using SYBR Green and a LightCycler^®^ 480 System (Roche Diagnostics K.K., Tokyo, Japan). Z‐linked *Tpi* was amplified using TPI‐F (5′–GGCCTCAAGGTCATTGCCTGT–3′) and TPI‐R (5′–ACACGACCTCCTCGGTTTTACC–3′), Z‐linked *Ket* was amplified using Ket‐F (5′–TCAGTTAAGGCTATTAACGCTCTG–3′) and Ket‐R (5′–ATACTACCTTTTGCGGTTACTGTC–3′), and autosomal *EF‐1α* was amplified using EF‐1F (5′–AAATCGGTGGTATCGGTACAGTGC–3′) and EF‐1R (5′–ACAACAATGGTACCAGGCTTGAGG–3′) (Kern et al. 2015). For each qPCR, a standard dilution series of PCR products (10^8^, 10^7^, 10^6^, 10^5^, 10^4^, and 10^3^ copies per microliter) was included in order to estimate the absolute copy numbers of the target sequence in the samples. To prepare standard samples, PCR products were gel‐excised and purified by Wizard^®^ SV (Promega). Copy numbers of the standard samples were estimated by the concentration measured by a spectrophotometer, considering that the molecular weight of a nucleotide is 309 g/mol. For each qPCR, two replicates were performed that delivered similar results. All qPCRs were performed using a temperature profile of 40 cycles of 95°C for 5 s, 60°C for 10 s, and 72°C for 10 s. The qPCR data were analyzed by the Absolute Quantification analysis using the Second Derivative Maximum method implemented in the LightCycler^®^ 480 Instrument Operator Software Version 1.5 (Roche).

### RT‐PCR

RNA was extracted from adult abdomens that were stored at –80°C using RNeasy^®^ Mini Kit (Qiagen, Tokyo, Japan). The cDNA synthesized by using Superscript™ III (Invitrogen) and Oligo(dT) was used as a template for RT‐PCR. A partial sequence of *dsx* that contains alternative splicing sites was amplified using a primer pair, E520F (5′–GCAACGACCTCGACGAGGCTTCGCGGA–3′) and EhdsxR4 (5′–AGGGGCAGCCAGTGCGACGCGTACTCC–3′) and a temperature profile of 94°C for 2 min, 30 cycles of 94°C for 1 min, 57°C for 1 min and 72°C for 1 min 30 s, followed by 72°C for 7 min. The sequences of seven *dsx^F^* isoforms and a *dsx^M^* isoform were deposited in DDBJ/EMBL/Genbank (LC215389‐LC215396).

## Results

### ALL‐FEMALE‐PRODUCING CF FEMALES HAVE A Z0 SEX CHROMOSOME CONSTITUTION

**Figure 1:**
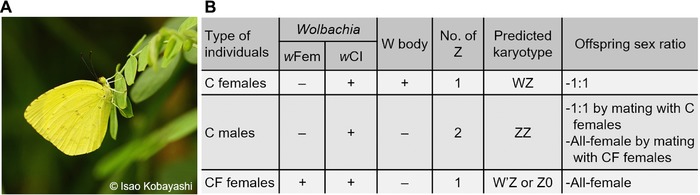
*E. mandarina* butterflies used in this study. (A) A photo of *E. mandarina* taken in Tanegashima Island. (B) Characteristics of three types of *E. mandarina* individuals inhabiting Tanegashima Island.

**Figure 2:**
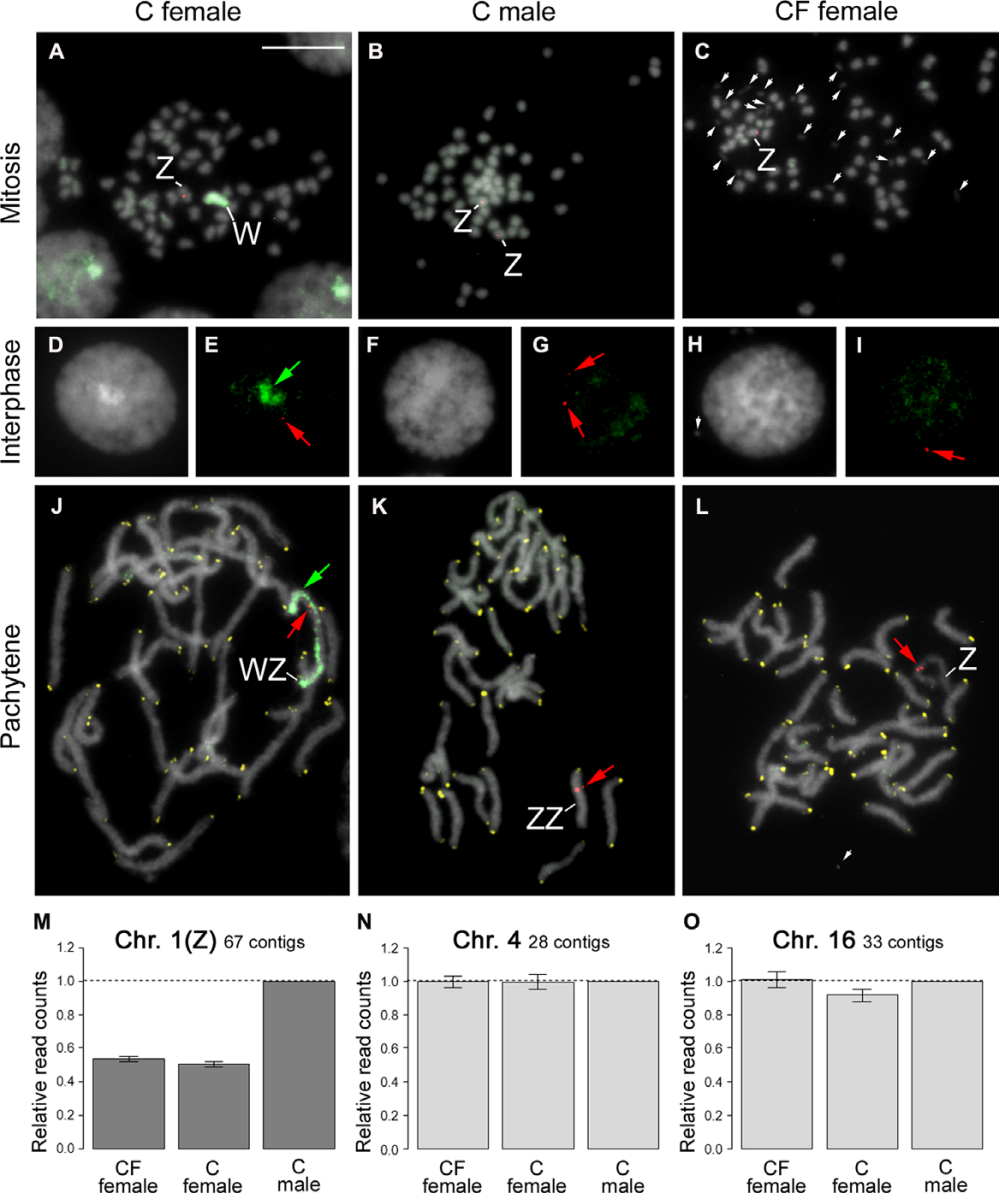
Fluorescence in situ hybridization and sequence read counts for a C female, C male, and CF female *E. mandarina*. (A–C) Mitotic complements hybridized with a genomic probe (green; green arrows) and a Z‐linked *Ket* probe (red; red arrows) in a C female (2n = 62) (A), C male (2n = 62) (B), and CF female (2n = 61) (C). (D–I) Genomic in situ hybridization (GISH) and FISH with a Z‐linked *Ket* probe performed on interphase nuclei of *E. mandarina* C females (D, E), C males (F, G), and CF females (H, I). (J–L) GISH, telomere‐FISH, and FISH with *Ket* probe performed on pachytene complements of *E. mandarina* C females (G, *n* = 31), C males (H, *n* = 31), and CF females (I, *n* = 31). Green paint signals in A, E, and J revealed that C females have the W chromosome. The *Ket* probe signals (red) appeared on the Z pairing to the W in C females (J), the ZZ bivalent in C males (K), and the Z univalent of CF females (L). The single signals were observed both in C and CF female nuclei. The signals in C females (J) and males (K) clearly showed their respective WZ and ZZ chromosome sets, and a Z0 chromosome set in CF females (L). W: W chromosome; Z: Z chromosome; white arrows: *Wolbachia*‐like structures. A bar represents 10 μm. M–O: Relative normalized sequence read counts in CF females, C females, and C males for 67 contigs homologous to *Bombyx mori* loci on chromosome 1 (Z chromosome; M), 28 contigs homologous to *B. mori* loci on chromosome 4 (N), and 33 contigs homologous to *B. mori* loci on chromosome 16 (O), with relative read counts set to 1 (males). Details about genome sequencing are provided in Materials and Methods.

We performed FISH on *E. mandarina* chromosomes prepared from CF females, C females, and C males collected on Tanegashima Island (Fig. 1; Fig. S1). In the mitotic complement of C females, which harbor a 2*n* = 62 karyotype, genomic probes highlighted the W chromosome, with scattered signals on the other chromosomes (Fig. 2A; see Materials and Methods for technical details). A probe for the Z‐linked gene *Kettin* (*Ket*) identified the single Z chromosome in C females (Fig. 2A), and also hybridized to the Z chromosome paired with the W chromosome in pachytene bivalents (Fig. 2J). The *Ket* probe identified two Z chromosomes in the mitotic complement of C males (Fig. 2B; 2*n* = 62). No painted W chromosome was observed in interphase nuclei (Fig. 2H,I), the mitotic complement (Fig. 2C), and pachytene complement (Fig. 2L) of CF females, but the *Ket* signal appeared on the single Z chromosome in the mitotic complement (Fig. 2C) and Z univalent in the pachytene complement (Fig. 2L). Based on the relative read counts homologous to *Bombyx mori* Z‐linked and autosomal genes in females and males, our genome sequencing data support the notion that CF and C females have one Z chromosome (Fig. 2M–O; Fig. S2), which is consistent with genomic qPCR data based on two loci, *Triosephosphate isomerase* (*Tpi*) and *Ket*, relative to the autosomal gene *EF‐1α* (Kern et al. 2015). Thus, our results directly reveal the sex chromosome constitution of C females, C males, and CF females as WZ, ZZ, and Z0, respectively. This confirms one of two previously suggested sex chromosome constitution of CF females (Kern et al. 2015) while it disproves another previous interpretation based on W‐body diagnosis that CF females are ZZ (Hiroki et al. 2002; Narita et al. 2007a).

### ALL EMBRYOS OVIPOSITED BY CF FEMALES ARE Z0

**Figure 3:**
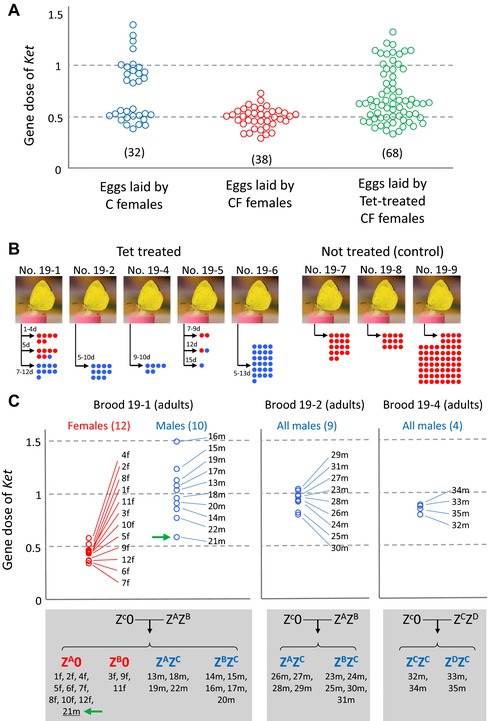
Effects of *w*Fem on Z‐linked gene dose in *E. mandarina* offspring. (A) Estimate of the gene dose of *Ket* (relative gene copies per copy of *EF‐1α*) by genomic quantitative polymerase chain reaction (qPCR) analysis in each of the fertilized eggs laid by C females, CF females, and tetracycline (tet)‐treated CF females. Each colored circle represents a single fertilized egg. Sample sizes are given in parentheses. (B) Offspring sex ratio of five females tet‐treated prior to oviposition and three nontreated CF females. Numbers to the left of the arrows represent duration (days) of tet treatment. Blue dots and red dots represent males and females, respectively. (C) Estimate of the gene dose of *Ket* (relative gene copies per copy of *EF‐1α*) by genomic qPCR in each of the adult offspring produced by CF females that were tet‐treated during the adult stage (prior to oviposition). Each circle represents an adult offspring. Z chromosomes of these offspring individuals were genotyped as Z^A^, Z^B^, Z^C^, or Z^D^ on the basis of intron nucleotide sequence of Z‐linked *Tpi*. The green arrow points to a male individual (adult) whose karyotype was considered to be Z0 but possibly ZZ’ (see text for details). f: female, m: male.

We performed real‐time genomic qPCR (to detect Z‐linked *Tpi* or *Ket* relative to autosomal *EF‐1α*) on individual fertilized eggs (48‐h embryos), and found that embryos oviposited by C females had either one or two Z chromosomes at nearly equal frequencies (Fig. 3A left; Fig. S3). In contrast, all embryos oviposited by CF females were single Z carriers (Fig. 3A middle; Fig. S3). These findings indicate that the progeny of CF females are exclusively Z0 individuals, supporting the previous data that the maternal Z chromosomes are not inherited in CF lineages (Kern et al. 2015).

### *WOLBACHIA* CAUSES THE EXCLUSIVE PRODUCTION OF Z0 EMBRYOS BY CF FEMALES

To abolish the effects of *Wolbachia*, tetracycline (tet) was administered to adult CF females previously inseminated by antibiotic‐treated male offspring of C females. The Z‐linked gene dose of embryos laid by these tet‐treated females ranged from approximately 0.5–1.0, indicating that some embryos are Z0 and others are ZZ (Fig. 3A right; Fig. S3). This suggests that the *Wolbachia* strain *w*Fem in CF females causes the exclusive production of gametes without sex chromosomes that then develop as Z0 embryos after fertilization. Therefore, our finding is the first empirical evidence that in a female‐heterogametic species the sex‐specific linkage disequilibrium can be caused by cytoplasmic elements (Hurst 1993; Beukeboom and Perrin 2014). Furthermore, *Wolbachia*‐like structures were observed near the chromosomes in CF females (Fig. 2C) while less apparent in C females (Fig. 2A) and C males (Fig. 2B), and this may represent different tropism and function of *w*Fem when contrasted with *w*CI. Sixty‐nine adults (15 females and 54 males) were obtained from offspring produced by five tet‐treated adult CF females (Fig. 3B). Three of these tet‐treated females produced only male offspring. Exclusive production of males was previously observed in tet‐treated *E. mandarina* females derived from a different population on Okinawa‐jima Island, Okinawa Prefecture, Japan (Hiroki et al. 2002). In this study, we obtained 15 female offspring from two broods in the first days after tet treatment; however, the mothers produced more males as the duration of tet treatment increased, and eventually produced only males. Examination of the Z‐linked gene dose of these offspring by genomic qPCR showed that the females had one Z chromosome, whereas almost all of the males had two Z chromosomes (Fig. 3C). The nucleotide sequences of the introns of the *Tpi* gene demonstrate that, in brood 19‐1, all females (*n* = 12) were hemizygous and nine out of 10 males were heterozygous (Fig. 3C; Fig. S4). Curiously, one male (21m) that exhibited the lowest gene dose of *Ket* (0.588) appeared to be hemizygous (Fig. 3C). These results suggest that the emerged females had a Z0 sex chromosome constitution, whereas most males had a ZZ sex chromosome constitution, with one exception (21m) of either Z0 or ZZ’ (Z’ represents partial deletion/mutation in Z). These results also demonstrate that, in principle, tet‐treated adult CF females can oviposit embryos with either a Z0 or ZZ sex chromosome constitutions (Fig. 3A right; Fig. S3). However, Z0 males appear to have zero or very low survival rates.

### INVOLVEMENT OF *WOLBACHIA* IN THE SEX DETERMINATION OF *E. MANDARINA*

**Figure 4:**
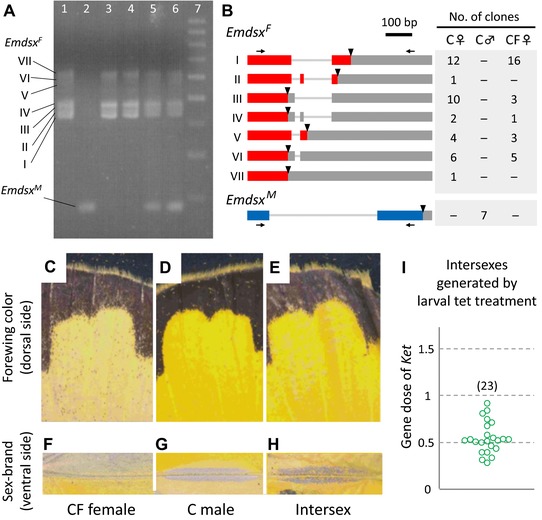
Effects of *w*Fem on splicing of the *doublesex* gene in *E. mandarina*. (A) Reverse‐transcription polymerase chain reaction (RT‐PCR) products of *E. mandarina doublesex* (*Emdsx*) run on an agarose gel. Lane 1: C female; lane 2: C male; lanes 3 and 4: CF females; lanes 5 and 6: intersexes generated by tetracycline (tet) treatment of larvae produced by CF females; lane 7: 100‐bp ladder. Females have at least seven splicing products, whereas males have a single product. (B) Structures of the splicing products of *Emdsx*. Translated regions are indicated by red and blue bars, untranslated regions by gray bars, and stop codons by triangles. Numbers of clones obtained by cloning the RT‐PCR products are shown in the table on the right. (C–H) color and morphology of forewings. Females are pale yellow on the dorsal side of the forewings (C) and do not have sex brand on the ventral side of the forewings (F), while males are intense yellow on the dorsal side of the forewings (D) and have sex brand on the ventral side of the forewings (G). Many of the intersexes generated by tet‐treating CF larvae are strong yellow on the dorsal side of the forewings (E) and have faint sex brand on the ventral side of the forewings (H). (I) Estimate of the gene dose of *Ket* (relative gene copies per copy of *EF‐1α*) by genomic qPCR in each of the intersex CF individuals that were produced by tet treatment during larval stages. Each circle represents an adult offspring.

Next, we fed CF larvae on a tet‐containing diet. As previously observed (Narita et al. 2007a), all individuals treated in this way developed an intersex phenotype at the adult stage, typically represented with male‐like wing color and an incomplete male‐specific structure on the wing surface (Fig. 4E,H; Fig. S6). The qPCR assay to assess the Z‐linked gene dose revealed that these intersexes (*n* = 23) had just one Z chromosome (Fig. 4I), and therefore a Z0 genotype. Because these Z0 individuals were destined to develop as females without tet treatment, *w*Fem is likely to be responsible for female sex determination. Further evidence in support of this idea was obtained by examining the sex‐specific splicing products of *dsx* (Fig. S7), a widely conserved gene responsible for sexual differentiation (Bopp et al. 2014). Similar to *B. mori* (Ohbayashi et al. 2001), C females exhibited female‐specific splicing products of *E. mandarina dsx* (*Emdsx^F^*), whereas C males had a male‐specific splicing product of *E. mandarina dsx* (*Emdsx^M^*; Lanes 1 and 2 in Fig. 4A, respectively; Fig. 4B). Similarly to C females, CF females exhibited exclusive expression of *Emdsx^F^* (Lanes 3 and 4 in Fig. 4A; Fig. 4B). Intersexual butterflies, generated by feeding the larval offspring of CF females a tet‐containing larval diet, expressed both *Emdsx^F^* and *Emdsx^M^* (Lanes 5 and 6 in Fig. 4A; Fig. S5).

## Discussion

We provide comprehensive and conclusive indirect (qPCR of Z gene dosage) and direct (W chromosome painting; genomic analyses) evidence for the absence of the W chromosome in CF individuals. Furthermore, we demonstrate that the *Wolbachia* strain *w*Fem is directly responsible for the disruption of sex chromosome inheritance in CF females of *E. mandarina*. This is the first empirical proof for previous theoretical predictions that cytoplasmic SGEs, such as *Wolbachia*, can disrupt chromosome inheritance (e.g., by meiotic drive). In *E. mandarina*, *w*Fem has a dual role in both causing disruption of maternal inheritance of Z chromosomes and converting male sex determination into female sex determination (feminization) in Z0 lineages that have lost W chromosome and its female‐determining function.

### *WOLBACHIA* DISRUPTS Z CHROMOSOME INHERITANCE IN Z0 FEMALES

**Figure 5:**
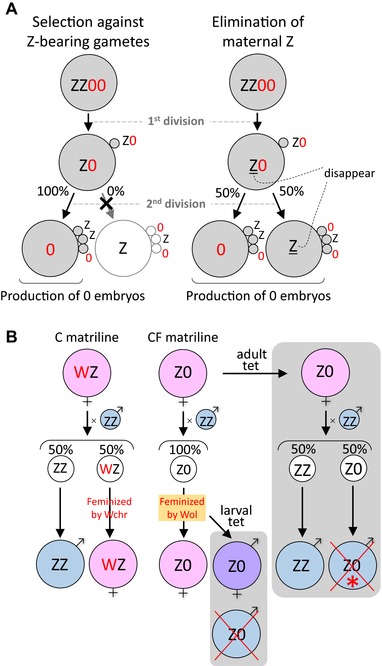
(A) Schematic illustration of two alternative mechanistic models of disruption of Z chromosome inheritance that explain the observed data. The “Selection against Z gametes” model assumes that Z‐bearing gametes are selected against during meiosis (left). The “Elimination of maternal Z” model assumes that Z chromosomes are eliminated during or after normal meiosis, while all the autosomes being intact (right). (B) All‐female production explained by *Wolbachia*–host interaction. Effects of *w*Fem on the development and sex determination of *E. mandarina*, and outcomes of larval versus adult tet treatment are illustrated. Asterisk: The majority of Z0 males die, but a few survived.

Our data provides evidence that the exclusive production of Z0 embryos by CF females is due to a yet unidentified developmental process that leads to the disruption of sex chromosome inheritance in CF females, thereby the absence of maternal Z chromosome in CF offspring (Kern et al. 2015). We believe that two mutually exclusive hypotheses can account for the disruption of Z chromosome inheritance observed in CF individuals (Fig. 5A). The first assumes that a gamete without the maternal Z chromosome (or without any sex chromosome overall), is always selected to become an egg pronucleus (meiotic drive against Z‐bearing gametes) (Fig. 5A left) (Pardo‐Manuel de Villena and Sapienza 2001). The second assumes that meiosis itself is normal, and that maternal Z chromosomes (or sex chromosomes in general), are selectively eliminated from Z‐bearing gametes during, or possibly after, meiosis (Fig. 5A right). At present, it is unclear which of the two scenarios (meiotic drive *sensu stricto* or elimination of the maternal Z) is more plausible. However, it is noteworthy that, in the moth *Abraxas grossulariata*, a matriline consisting of putative Z0 females was observed to produce only females or a great excess of females, and the underlying mechanism was considered to be the selective elimination of Z chromosomes (Doncaster 1913, 1914, 1915, 1922). However, the presence of cytoplasmic bacteria such as *Wolbachia* has not yet been examined for this moth species. If we assume that the elimination of the maternal Z chromosome occurs in *E. mandarina*, the exceptional individual 21m (Fig. 3C) could be viewed as ZZ’ rather than Z0, wherein Z’ is a maternal Z chromosome that was only partially deleted in the position including *Tpi* and *Ket* by the incomplete action of *w*Fem. It is possible to further speculate that the presence of *w*Fem results in the elimination of sex chromosomes in general (Z or W chromosomes) and, therefore, the absence of W chromosomes in CF females may also be a direct effect of *w*Fem.

### THE FEMINIZING EFFECT OF *WOLBACHIA* COMPENSATES FOR THE LOSS OF THE W CHROMOSOME IN Z0 INDIVIDUALS

In general, lepidopteran species with Z0/ZZ sex chromosome constitution are considered to determine their sexes by Z‐counting mechanisms, wherein ZZ is male and Z0 is female (Traut et al. 2007; Sahara et al. 2012). However, the appearance of the male phenotype in Z0 individuals of *E. mandarina* after antibiotic treatment suggests that *w*Fem in Z0 individuals compensates for the loss of W and its female‐determining function (Fig. 5B). We speculate that the W chromosome of *E. mandarina* acts as an epistatic feminizer. In *B. mori*, the W chromosome–more specifically, a piRNA located on the W chromosome–acts as an epistatic feminizer by silencing *Masculinizer* on the Z chromosome (Kiuchi et al. 2014).

Reduced survival of Z0 individuals or their offspring after antibiotic treatment of larvae or adults, respectively, may suggest improper dosage compensation in Z0 males. Improper dosage compensation was also proposed to be the cause of male‐ and female‐specific lethality in *Wolbachia*‐infected and cured lines of *Ostrinia* moths (Kageyama and Traut 2004; Sugimoto and Ishikawa 2012; Fukui et al. 2015; Sugimoto et al. 2015). Another symbiont *Spiroplasma poulsonii* kills *Drosophila* males by targeting the dosage compensation machinery (Veneti et al. 2005; Cheng et al. 2016). It was recently found that *S. poulsonii* causes DNA damage on the male X chromosome interacting with the male‐specific lethal (MSL) complex. The DNA damage leads to male killing through apoptosis via p53‐dependent pathways (Harumoto et al. 2016).

### HOW DID THE COORDINATED DUAL EFFECTS OF *WOLBACHIA* EVOLVE?

**Figure 6:**
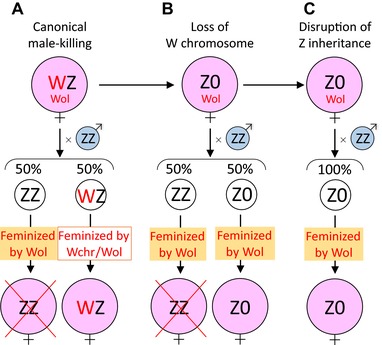
Hypothetical evolutionary trajectory of the *Wolbachia*–host interaction in *E. mandarina*. See Discussion for details.

We demonstrated that *w*Fem causes the disruption of Z chromosome inheritance and the conversion of sex determination in *E. mandarina* in two steps (Fig. 5B). This is similar to the dual role of *Wolbachia* and *Cardinium* in haplodiploid parasitoid wasps where they induce thelytokous parthenogenesis in a two‐step mechanism, comprising diploidization of the unfertilized egg followed by feminization (Giorgini et al. 2009; Ma et al. 2015). Here, we develop the potential evolutionary scenario that led to the appearance of both effects in *E. mandarina* (Fig. 6).

A WZ female *Eurema* butterfly may have acquired *w*Fem that exerted a feminizing effect on ZZ males. The feminizing effect was lethal to ZZ individuals because of improper dosage compensation, as evident in *Wolbachia*‐infected *Ostrinia* moths (Fig. 6A) (Fukui et al. 2015; Sugimoto et al. 2015). This could be viewed as a manipulation similar to a male‐killing phenotype (Dyson et al. 2002; Harumoto et al. 2016). However, the feminizing effect of *w*Fem was redundant in WZ females where the W chromosome acted as a female determiner (Kiuchi et al. 2014). Conversely, the function of W had also become redundant in CF individuals and this could have led to the loss of the W chromosome and the rise of a Z0 lineage (Fig. 6B). Similarly, in *Ostrinia* moths, a female‐determining function is thought to have been lost from the W chromosome in *Wolbachia*‐infected matrilines (Sugimoto and Ishikawa 2012). Spontaneous loss of a nonfunctional W chromosome may be easier than expected: in a wild silkmoth *Samia cynthia*, the W chromosome does not have a sex‐determining function, and Z0 females are frequently obtained in experimental crosses between subspecies (Yoshido et al. 2016). *Wolbachia* has previously been found to be associated with the loss and birth of W chromosomes in the woodlouse *Armadillidium vulgare* (Rigaud et al. 1997; Leclercq et al. 2016). However, in *A. vulgare* it has not yet been tested whether *Wolbachia* interferes with chromosome segregation and inheritance as we have mechanistically demonstrated it for *E. mandarina*; that is after the loss of the W chromosome in CF lineages, *Wolbachia* then acquired a novel function that affected female oogenesis and resulted in the disruption of Z chromosome inheritance (Fig. 6C). It is unlikely that the disruption arose prior to the appearance of female‐determining function of *Wolbachia*: if the appearance of the disruption of Z chromosome inheritance were to precede the loss of the W chromosome, the feminizing or female‐determining function would become unnecessary for *Wolbachia* because there would be no males.

In the short term, disruption of Z chromosome inheritance in females in a female‐heterogametic species represents a great advantage to cytoplasmic symbionts because all vertically transmitted symbionts gain the opportunity to survive. However, males are still required for fertilization, and fixation of the symbionts in the host population will inevitably lead to the extinction of both the symbionts and the hosts (Hatcher et al. 1999). In the long term, suppressors against sex ratio distortion, as has been observed for the male‐killing phenotypes in the butterfly *Hypolimnas bolina* or a ladybird beetle (Charlat et al. 2007; Majerus and Majerus 2010), can be expected to evolve in the host. However, the evolutionary outcomes of the suppression of a combined action of *Wolbachia* in *E. mandarina* would be different from that of male‐killing suppression, because it would lead to all‐male progeny, resulting in the loss of the matriline that inherits the feminizing and sex‐distorting *Wolbachia*. This process thereby selects for an increased frequency of WZ females.

### CONCLUDING REMARKS

In summary, we demonstrate for the first time that the manipulation of sex chromosome inheritance and cytoplasmically induced disruption of chromosome inheritance, which would either be the result of meiotic drive against Z‐bearing gametes or elimination of Z chromosome, can be added to the repertoire of host manipulations induced by *Wolbachia*. Therefore, the host effects of this bacterium are far more diverse and profound than previously appreciated. Disentangling these complex interactions between insects and *Wolbachia* may provide further exciting discoveries in the areas of host–parasite interactions, endosymbiosis as well as cell and chromosome biology in years to come, and perhaps also provide new avenues for pest population control.

Associate Editor: Jon Slate

## Supporting information


**Figure S1**. Distribution of *E. mandarina* in Japanese archipelago.

**Figure S2**. Relative normalized sequence read counts for 440 contigs of *E. mandarina* that matched to *B. mori* loci on 28 chromosomes.

**Figure S3**. Estimate of Z‐linked gene dose of *E. mandarina*.

**Figure S4**. Genotyping of Z chromosome based on nucleotide polymorphism of *Tpi*.

**Figure S5**. Detection of *Emdsx* in adults that were tet‐treated during various larval stages.

**Figure S6**. Intersexual and normal adults.

**Figure S7**. Amino acid sequences of *dsx* genes.
