# Supplementary material for: Feminizing Wolbachia endosymbiont disrupts maternal sex chromosome inheritance in a butterfly species
Source: Evol Lett. 2017 Oct 31;1(5):232–44. doi: 10.1002/evl3.28 (PMC6121850; doi:10.1002/evl3.28)
Supplement: Supplementary file 1 — Figure S1. Distribution of E. mandarina in Japanese archipelago. [file EVL3-1-232-s001.pdf]

A

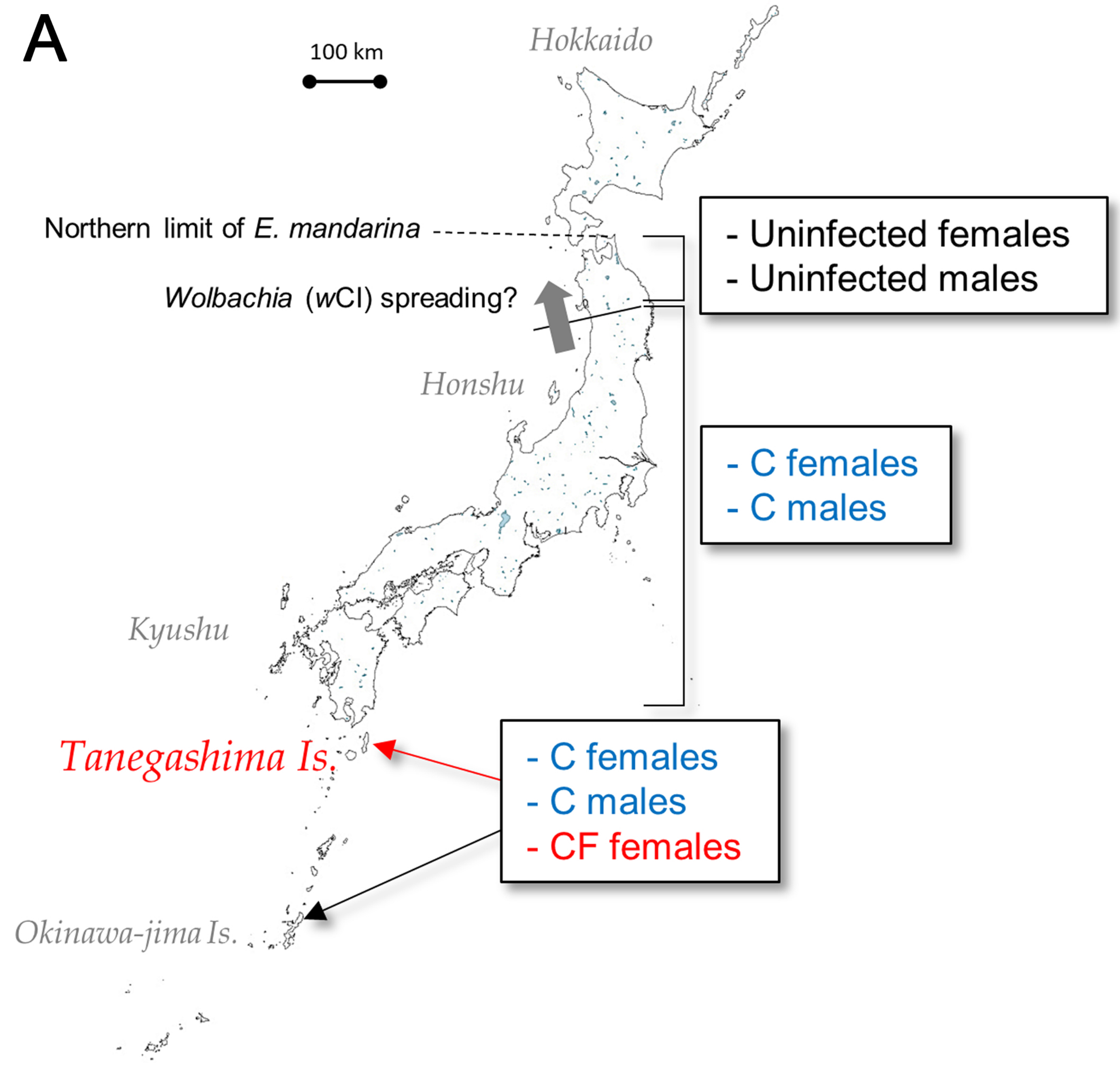

B

Type of individuals	*Wolbachia* infection status	Offspring sex ratio	Distribution
Uninfected females and males	–	1:1	Northern Honshu
C females and males	*w*CI	1:1	Everywhere except northern Honshu
CF females	*w*CI and *w*Fem	all-female	Found in Tanegashima and Okinawa-jima Islands
