# Supplementary figures and images for: Feminizing Wolbachia endosymbiont disrupts maternal sex chromosome inheritance in a butterfly species
Source: Evol Lett. 2017 Oct 31;1(5):232–44. doi: 10.1002/evl3.28 (PMC6121850; doi:10.1002/evl3.28)

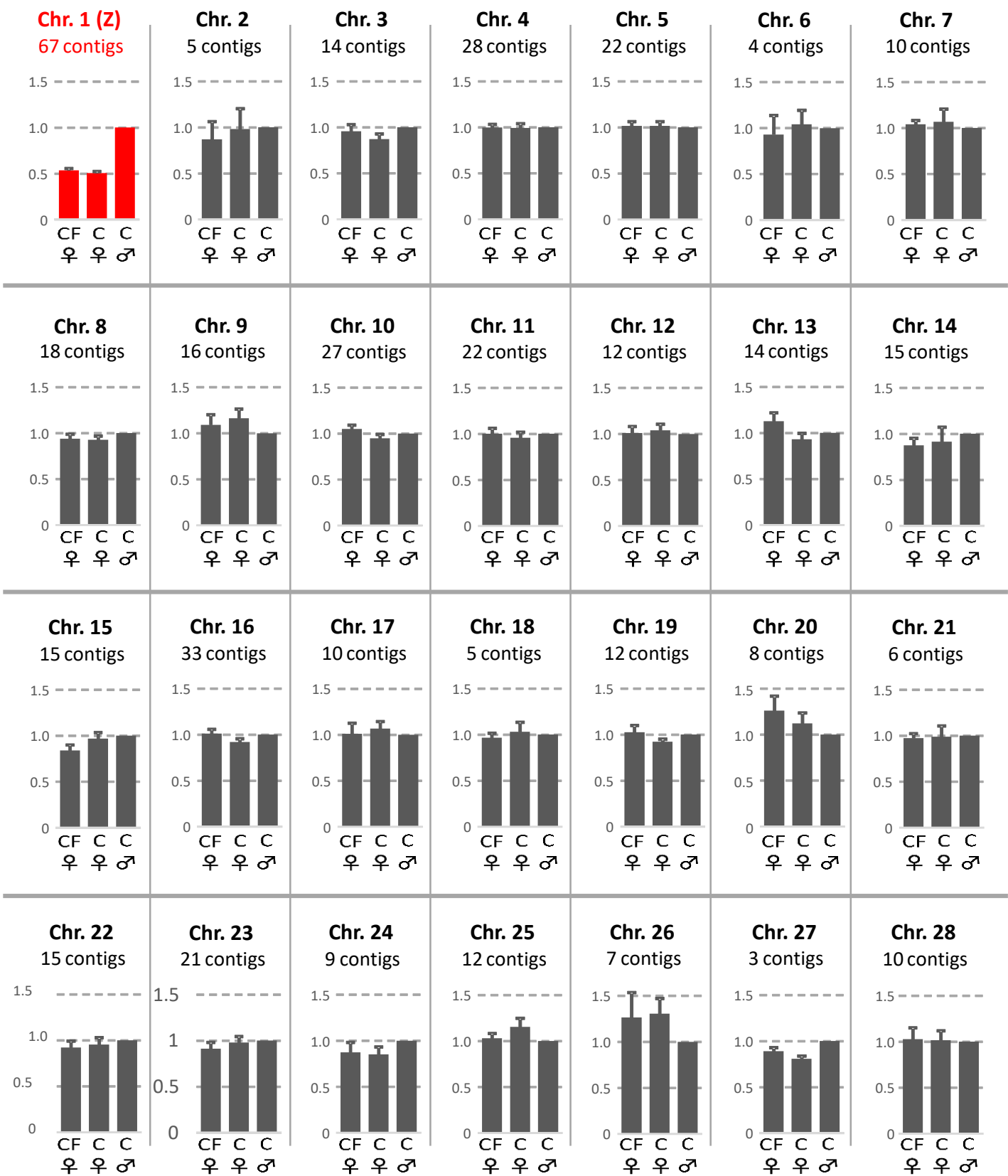

Supplement: Supplementary file 2 — Figure S2. Relative normalized sequence read counts for 440 contigs of E. mandarina that matched to B. mori loci on 28 chromosomes. [file EVL3-1-232-s002.pdf]

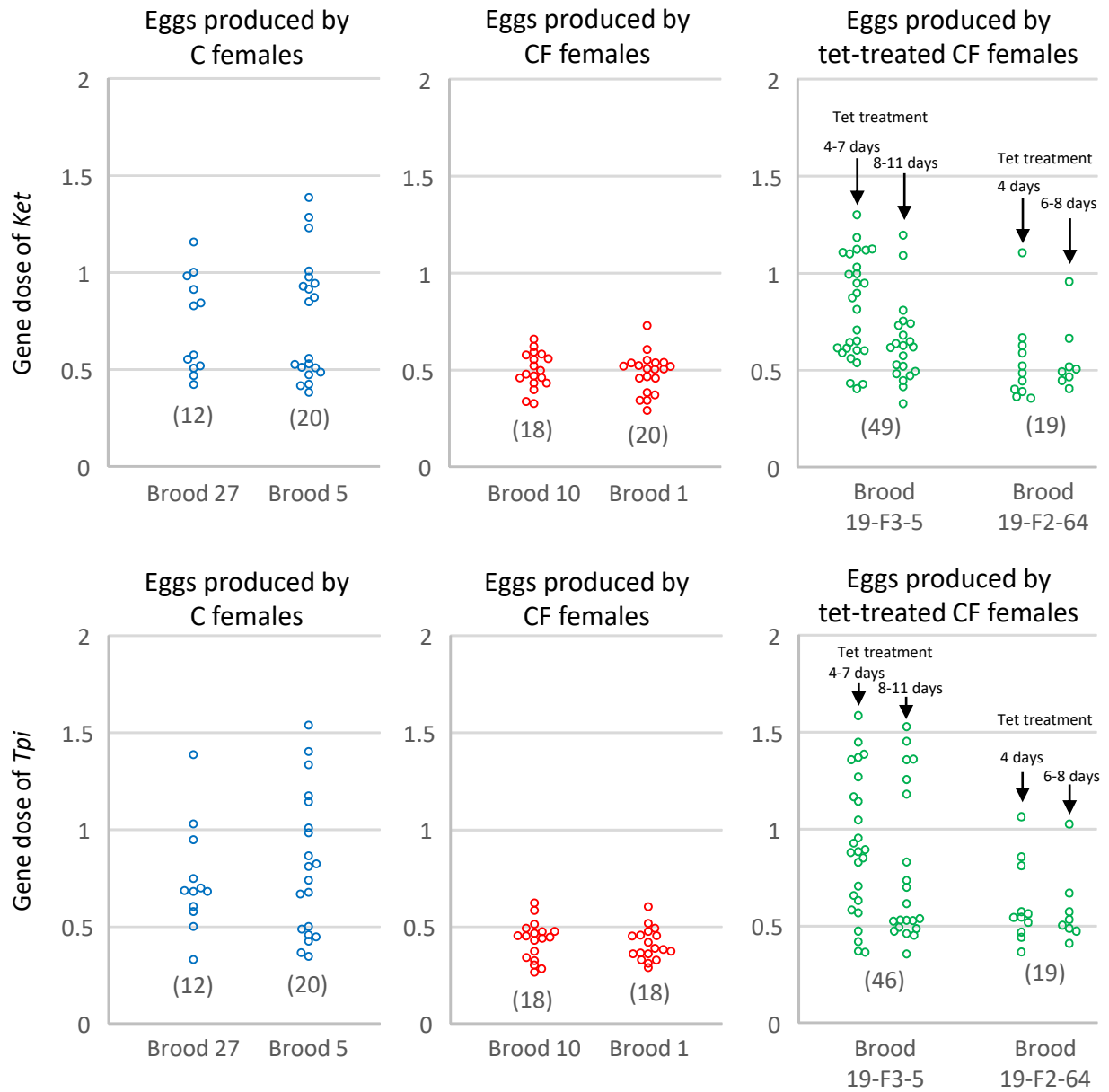

Supplement: Supplementary file 3 — Figure S3. Estimate of Z‐linked gene dose of E. mandarina. [file EVL3-1-232-s003.pdf]

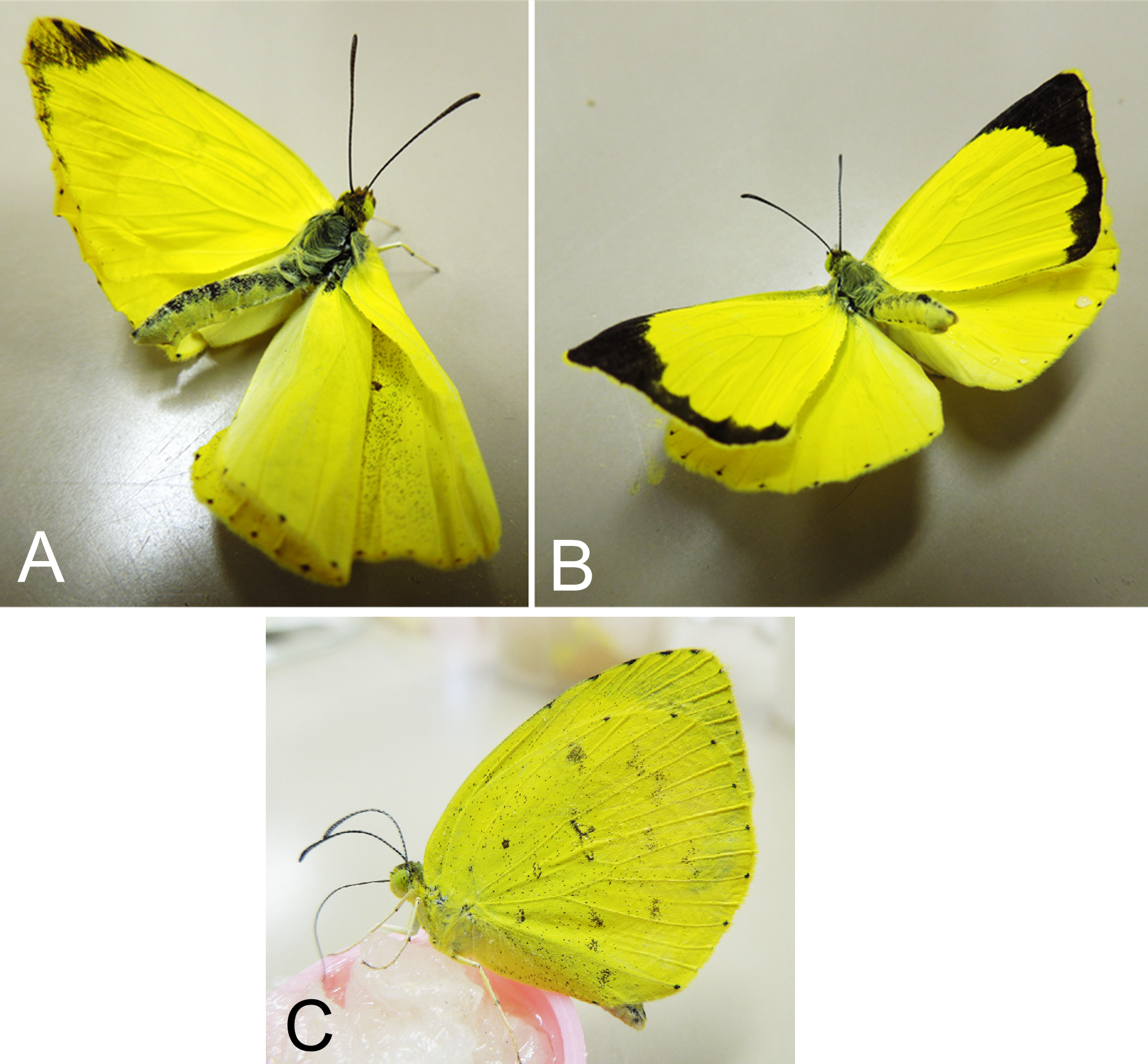

Supplement: Supplementary file 6 — Figure S6. Intersexual and normal adults. [file EVL3-1-232-s006.tif]

A

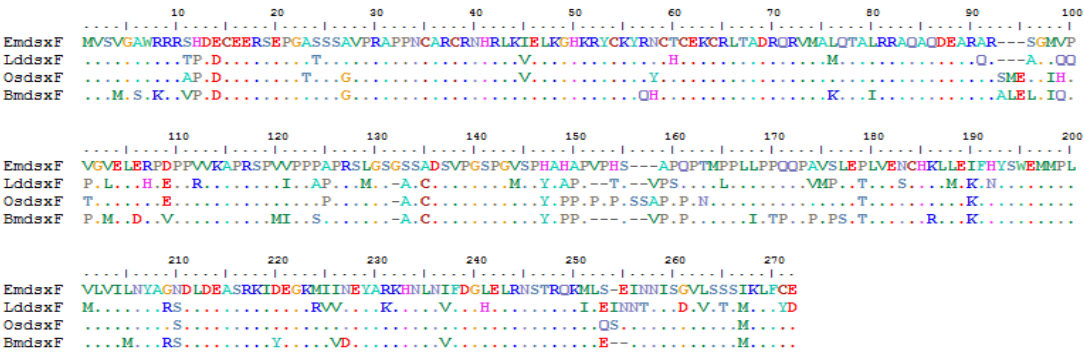

B

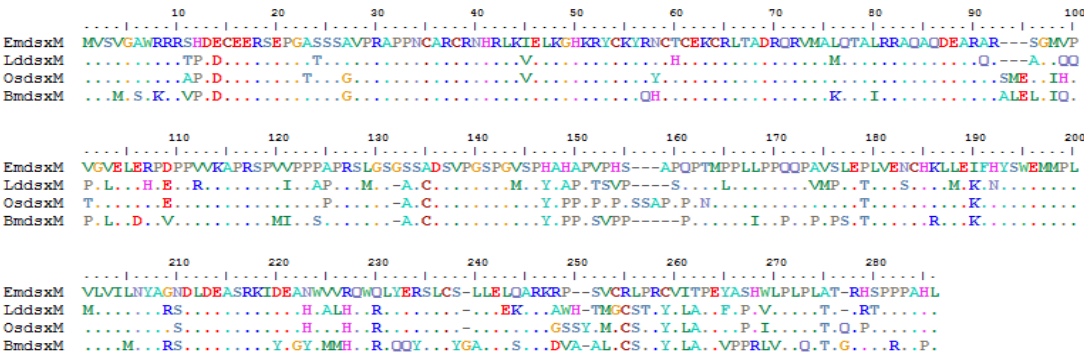

C

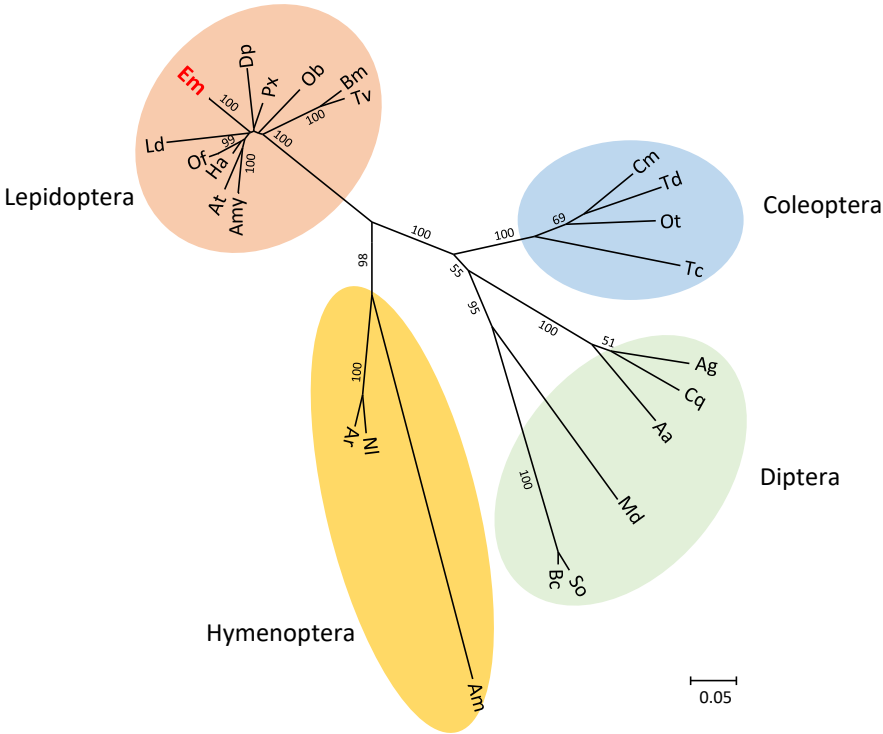

Supplement: Supplementary file 7 — Figure S7. Amino acid sequences of dsx genes. [file EVL3-1-232-s007.pdf]
