# Supplementary material for: Feminizing Wolbachia endosymbiont disrupts maternal sex chromosome inheritance in a butterfly species
Source: Evol Lett. 2017 Oct 31;1(5):232–44. doi: 10.1002/evl3.28 (PMC6121850; doi:10.1002/evl3.28)

A Sequence polymorphism of Z-linked *Tpi* gene

Genotype of Z chromosome	Polymorphic sites													
	109	110	111	112	135	157	160	173	175	228	233	324	403	425
Z^A	A	T	G	G	A	A	T	C	T	T	T	T	C	G
Z^B	–	–	–	–	A	A	C	T	T	A	T	–	T	G
Z^C	A	T	G	G	G	A	T	C	T	T	T	T	T	G
Z^D	A	T	G	G	A	G	C	T	A	T	G	T	T	A

B Examples of genotyping of sex chromosomes based on sequence data

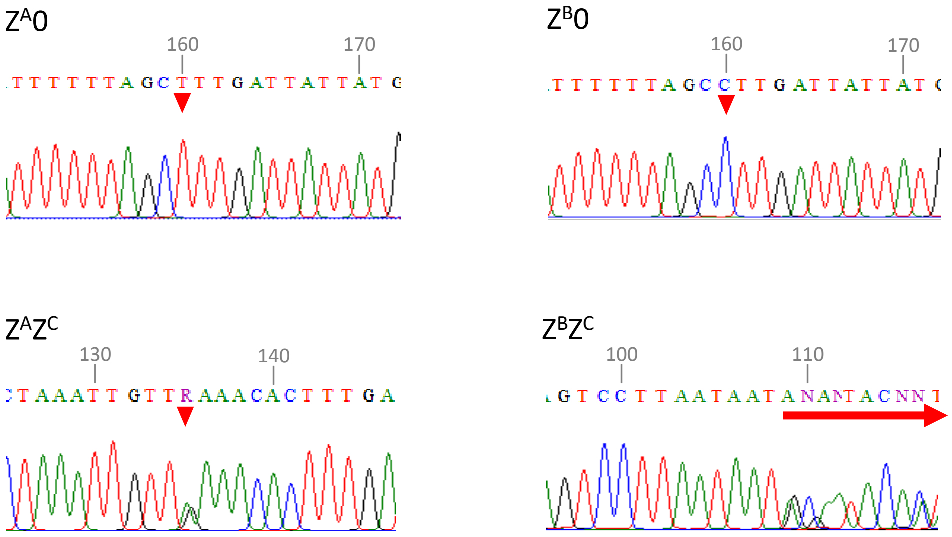

Supplement: Supplementary file 4 — Figure S4. Genotyping of Z chromosome based on nucleotide polymorphism of Tpi. [file EVL3-1-232-s004.pdf]
