# Supplementary material for: Feminizing Wolbachia endosymbiont disrupts maternal sex chromosome inheritance in a butterfly species
Source: Evol Lett. 2017 Oct 31;1(5):232–44. doi: 10.1002/evl3.28 (PMC6121850; doi:10.1002/evl3.28)

No. of adult offspring produced by CF females

A Non treated

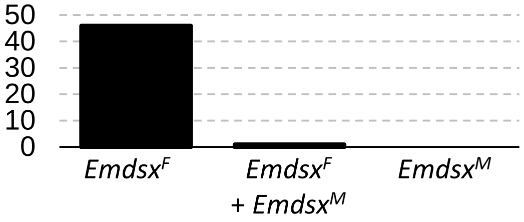

B Tet treated (during 4th to 5th instar larval stages)

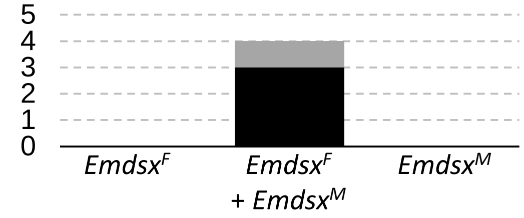

C Tet treated (during 3rd to 5th instar larval stages)

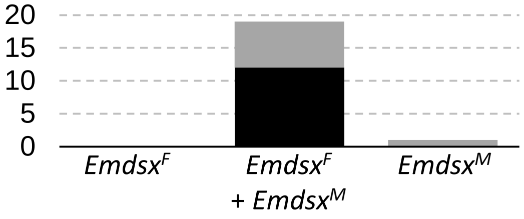

D Tet treated (during 2nd to 5th instar larval stages)

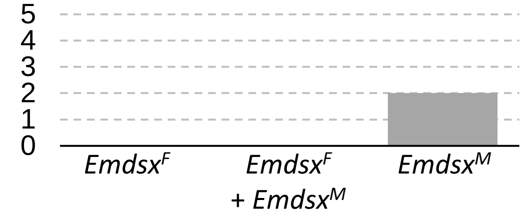

E Tet treated (during 1st to 5th instar larval stages)

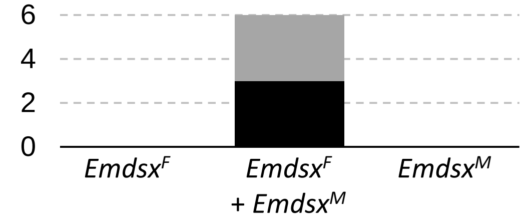

No. of adult offspring produced by C females

F Non treated

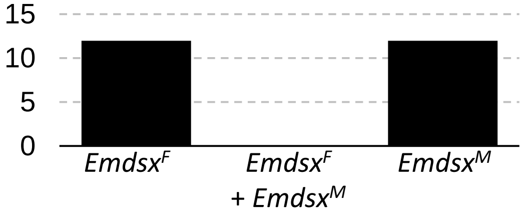

Supplement: Supplementary file 5 — Figure S5. Detection of Emdsx in adults that were tet‐treated during various larval stages. [file EVL3-1-232-s005.pdf]
